# Deformable image registration accuracy: impact of user-defined parameter selection on contour propagation for deep inspiration breath-hold and free breathing breast radiotherapy

**DOI:** 10.1186/s12880-026-02447-4

**Published:** 2026-06-05

**Authors:** Stefan Knippen, Lea Pargmann, Steffen Weimann, Guido Hildebrandt, Kai Joachim Borm, Marciana Nona Duma

**Affiliations:** 1 Department of Radiotherapy and Radiation Oncology, University Hospital Jena, Friedrich Schiller University, Kastanienstraße 1, 07747 Jena, Germany; 2 Department of Radiation Oncology, Helios Clinics Schwerin - University Campus of MSH Medical School Hamburg, Helios Kliniken Schwerin, Schwerin, Germany; 3 Department of Radiation Oncology, University Medical Center Rostock, Rostock, Germany; 4 Department of Radiation Oncology, TUM School of Medicine and Health, TUM University Hospital, Klinikum rechts der Isar, Munich, Germany; 5 Department for Human Medicine, MSH Medical School Hamburg, Hamburg, Germany

**Keywords:** Deformable image registration, Breast cancer radiotherapy, Deep inspiration breath-hold, ANACONDA algorithm, Dice similarity index, Focus region selection, Contour propagation, Clinical target volume

## Abstract

**Purpose:**

Deformable Image Registration (DIR) is increasingly used in breast radiotherapy planning, but the impact of different parameter settings on clinical accuracy remains unclear. This study systematically evaluates how focus region selection and registration direction affect DIR performance, providing evidence-based recommendations for clinical implementation.

**Materials and methods:**

We analyzed 73 patients with left-sided breast cancer who underwent both free breathing (FB) and deep inspiration breath-hold (DIBH) planning CT scans. Clinical target volume (CTV), heart, left anterior descending artery, and both lungs were retrospectively contoured by one radiation oncologist on both datasets. Deformable Image Registration was performed bidirectionally (FB↔DIBH) using four different approaches: no focus region, CTV-focused, heart-focused, and surgical clip-focused registration. Volume differences and Dice Similarity Index (DSI) were calculated to assess registration accuracy. Generalized estimating equations analyzed possible correlations.

**Results:**

Focus region selection critically impacted DIR accuracy. CTV-focused registration achieved highest CTV overlap (DSI: 0.96 vs. 0.91 without focus, *p* < 0.005) but compromised other structures. Heart-focused registration optimized cardiac structure accuracy but reduced CTV precision. Registration direction showed minimal impact on volume differences but affected DSI values. Tidal volume significantly affected both CTV (*p* < 0.005) and heart DSI (*p* < 0.007), with opposite effects on each structure. Manual contour adjustment remained necessary in all cases regardless of parameter selection.

**Conclusion:**

DIR performance in breast radiotherapy is highly dependent on user-defined parameters, with no single configuration optimizing all structures simultaneously. Focus region selection creates a trade-off between local accuracy and global registration quality. Clinical implementation requires structure-specific parameter optimization and mandatory manual review. These findings establish practical limitations and benefits of hybrid DIR algorithms for breast radiotherapy workflows and highlight the continued necessity of expert oversight.

## Introduction

Breast cancer is the most common cancer in women, and with improved treatments and increasing long-term survivorship, minimizing late treatment effects has become a clinical priority [1, 2]. Radiation therapy and treatments like trastuzumab and anthracyclines can cause cardiac issues. Trastuzumab can lead to left ventricular dysfunction and chronic heart failure, while anthracyclines can result in myocardial damage and potentially irreversible chronic heart failure [3–5]. Postoperative breast cancer radiation therapy can also have dose-dependent cardiac side effects, with an increased risk of major cardiac events associated with mean cardiac dose, manifesting clinically many years after treatment [6, 7].

While modern radiation therapy techniques have improved the therapeutic ratio, further optimization of heart-sparing approaches remains essential [8]. Deep inspiration breath-hold (DIBH) is a widely studied method for sparing the heart during radiation therapy, but its benefits can vary based on patient anatomy and lung capacity [9, 10]. Not all patients may benefit from DIBH, and finding efficient ways to identify suitable candidates is essential. Gaal et al. demonstrated that approximately one in five patients are not suitable candidates for treatment in DIBH [11].

Many radiation oncology departments acquire both free breathing and DIBH CT datasets to evaluate the potential benefit of breath-hold treatment for individual patients. However, this evaluation process remains largely subjective, relying on physician judgement of anatomical factors and estimated dosimetric benefits.

Deformable image registration (DIR) has been proposed as a tool to standardize and improve this decision-making process between free breathing (FB) and DIBH approaches [12]. DIR has been validated across multiple anatomical sites [13–15] and has been proposed as a tool to standardize decision-making between FB and DIBH approaches in breast cancer.

While approaches for automated segmentation show promise, both DIR and artificial intelligence (AI) based segmentation address different problems in this context. AI contouring applied independently to two datasets introduces inter-application variability that may confound pairwise FB-DIBH comparison. DIR propagates a contour between datasets, which may reduce this specific source of variability. However, DIR algorithms are themselves model-dependent, and different parameter settings - as demonstrated in this study - can yield substantially different results. The findings of this study are therefore specific to the RayStation^®^ ANACONDA (ANAtomically CONstrained Deformation Algorithm) algorithm and should not be interpreted as a generalizable methodological advantage of DIR over alternative approaches. However, in breast cancer, initial research more than a decade ago showed that using a breast template with DIR improved contouring consistency and efficiency compared to manual contouring, though clinical implementation remained limited [16].

In a 2015 study, Joo et al. analyzed breast cancer patients treated with DIBH radiation therapy and found significant dose reduction to the left anterior descending artery (LAD), resulting in a lower estimated risk of coronary events with DIBH. They applied DIR to generate a deformation vector field, which was subsequently used to propagate clinical target volume (CTV) contours between datasets, but the impact of registration parameters on accuracy was not systematically evaluated [12].

Therefore, this study systematically evaluates the clinical performance of RayStation’s ANACONDA algorithm for left-sided breast cancer planning with particular focus on user-defined parameter optimization. Existing studies have not systematically evaluated how user-defined parameters affect DIR performance, creating uncertainty about optimal implementation strategies for clinical practice. In our clinical practice, both datasets are routinely acquired to evaluate the potential cardiac benefit of DIBH treatment, as well as a fallback for patients who cannot adhere to the necessary breathing protocol. Currently, this requires manual contouring on both datasets. A potential DIR-enhanced workflow would involve: (1) initial contouring on the intended treatment dataset, (2) DIR to propagate contours to the comparison dataset, and (3) dosimetric evaluation to determine optimal treatment approach. This clinical context explains why bidirectional DIR evaluation (FB↔DIBH) is essential for reliable contour propagation in either workflow scenario. Specifically, we addressed three critical questions that remain unanswered in current literature:

1. How do focus region selection and registration direction affect DIR accuracy?
2. What is the quantitative impact of these parameters on structure overlap using standardized metrics?
3. Do patient-specific factors influence DIR performance, and can these relationships guide clinical implementation?

## Materials and methods

For this retrospective analysis, we included patients who underwent treatment for left-sided breast cancer after breast-conserving surgery between 2017 and 2019. To be included in the study, patients had to have both a deep inspiration breath-hold and a free breathing planning CT dataset acquired during the same planning session without the patients getting up during the scans. The CT images had a slice thickness of 2.5 mm. Patients who underwent mastectomy or had breast implants were excluded from the analysis. Seventy-three patients were included in the study. All patients had no indication for nodal irradiation; therefore, the CTV comprised exclusively the left breast volume without nodal target sub-volumes. During the analysis, the CTV of the breast was contoured according to the ESTRO guideline [17], together with the heart, the left anterior descending artery (LAD) [18] and both lungs.

**Fig. 1:**
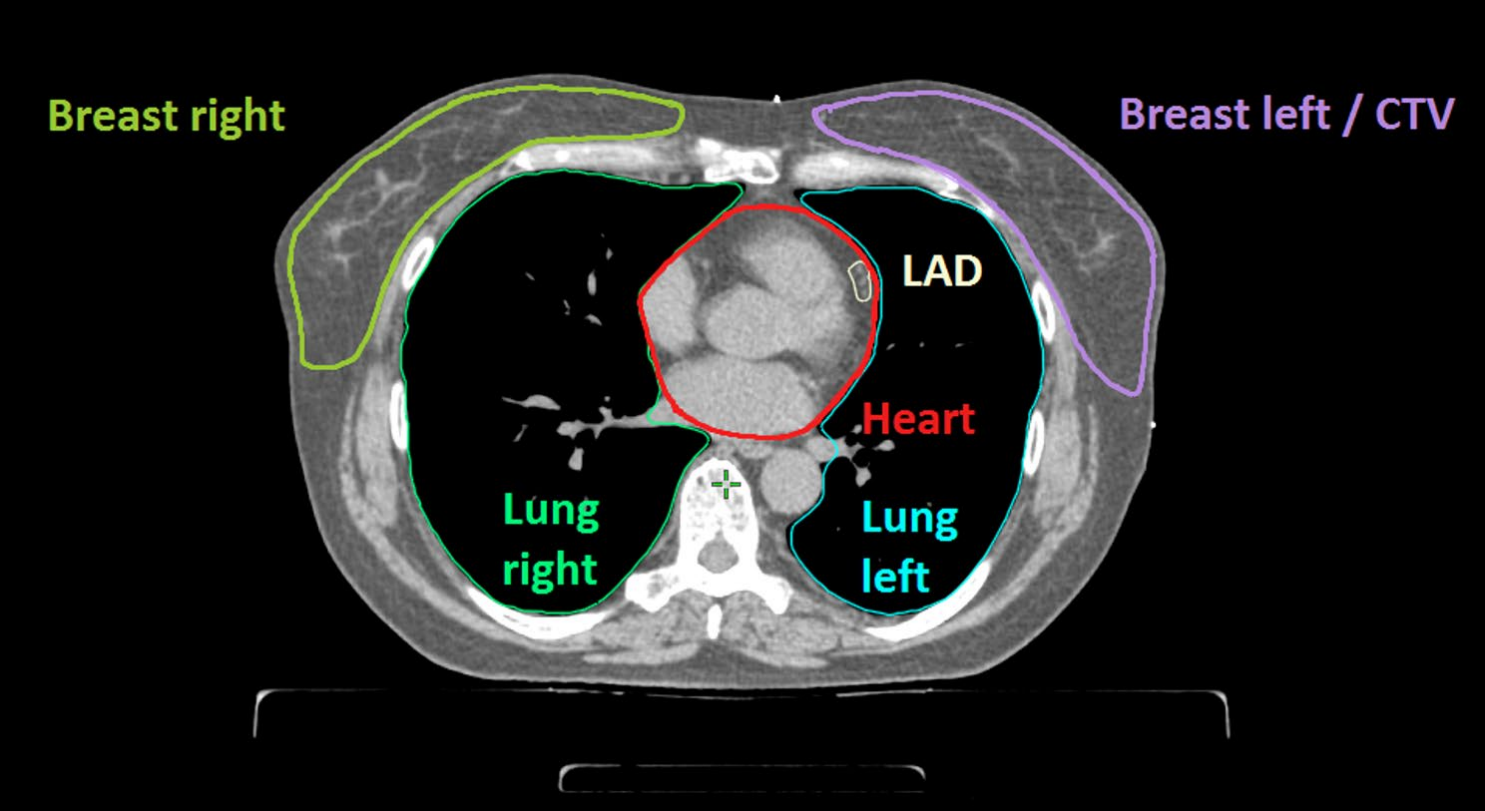
Example of contoured ROIs; Organs at Risk: Breast right, Lung right, Lung left, Heart, Left Anterior Descending Artery (LAD); Clinical Target Volume (CTV) = Breast left

Lung structures were generated using automated thresholding algorithms, while all other structures were manually contoured. Contouring of all datasets, including FB-CT and DIBH-CT, was performed by the same experienced radiation oncologist to reduce variability. Figure 1 shows contoured ROIs as an example.

Patients were grouped according to the breast volume as defined by McGhee et al. [19], where the “<399 ml” subgroup had volumes less than 399 ml, equivalent to cup size 12D AUS or 75D EUR. DIR was performed in the department’s treatment planning system (TPS) used for daily routine practice (RayStation^®^ - V.8 RaySearch Laboratories, Stockholm, Sweden). RayStation^®^ uses a hybrid approach using the ANACONDA algorithm, which is a hybrid algorithm that combines intensity-based (Hounsfield Units, HU) and geometric information. DIR was performed using default settings with a grid resolution of 0.25 × 0.25 × 0.25 cm. The default algorithm weights as reported by Weistrand and Svensson [20] are α = 1.0, γ = 0.5, δ = 0.5, and β = 400, with optimization performed on three resolution levels (10, 5, and 2.5 mm). The Focus ROI function was used to guide registration toward selected structures. Focusing on specific ROIs as control structures guides the deformation algorithm toward the selected structure, at the cost of potentially compromising registration accuracy in non-focused regions [21–23].

Prior to each DIR, automatic rigid image registration was performed using the thoracic spine as anatomical reference, without manual landmark placement. Deformable image registration was then performed. The optimization process aimed to minimize a mathematical function consisting of several terms. These terms included image similarity, shape-based regularization, grid regularization, and a penalty term applied when using control or focusing structures. Users could select regions of interest (ROIs) as control structures to guide registration, deforming them to align with target structures [20].

As mentioned above, ROIs were manually contoured in FB-CT and DIBH-CT by a radiation oncologist (mROI) and then automatically deformed to their counterpart (aROI) by the TPS algorithm.

We performed bidirectional DIR: from FB to DIBH and vice versa, to evaluate whether registration direction affects contour propagation accuracy and to inform the choice of primary contouring dataset in clinical practice. Although RayStation allows simultaneous selection of multiple focus ROIs, separate DIR runs were performed for each focus region to systematically isolate the effect of individual parameter choices, thereby disentangling the contribution of each focus region to overall registration accuracy.

Initially, we performed DIR without specific endpoints, using all CT dataset information. Later, we focused on specific ROIs as control structures: CTV, heart, and surgical clip region in the surgical cavity (contoured as a single composite ROI) after breast-conserving surgery, representing three different focus approaches for guiding the deformation algorithm. We quantified volume congruence using the Dice Similarity Index (DSI) [24], which ranges from 0 to 1. An index of 1 indicates complete overlap, while 0 indicates no overlap. Higher values indicate greater overlap and agreement between volumes, providing a numerical measure of similarity. Percentage volume differences were calculated as ∆V (%) = (aROI − mROI) / mROI × 100, where aROI is the volume of the automatically deformed structure and mROI the volume of the manually contoured reference structure.

DSI is calculated by:

$$\:DSI\:\:=\frac{2\mathrm{x}\mathrm{C}\mathrm{V}}{\mathrm{a}\mathrm{R}\mathrm{O}\mathrm{I}+\mathrm{m}\mathrm{R}\mathrm{O}\mathrm{I}}$$

where CV is the intersection volume between aROI and mROI, aROI is the volume of the automatically deformed ROI generated by DIR, and mROI is the volume of the manually contoured ROI.

DSI was chosen as the primary metric to ensure comparability with existing DIR literature in breast radiotherapy, where it remains the predominant validation metric. Registration quality was additionally assessed visually in a two-color overlay mode on the TPS.

### Statistics

Initial statistical analyses were performed in SPSS V27.0 (IBM^®^, Armonk, NY, USA) in collaboration with an independent biostatistics institute. Analyses of CTV enlargement magnitude were performed in R version 4.2.2, using the packages geepack (version 1.3.10) for GEE analysis and ggplot2 (version 3.4.0) for visualization. The analysis of the overlap of the studied ROI volumes was performed by using the DSI. Tidal volume was calculated by subtracting the measured lung volume in DIBH from the measured volume in FB [25]. From a physiological point of view this gives the tidal volume and the inspiratory reserve volume. As this study was exploratory and hypothesis-generating in nature, no formal power calculation was performed and no correction for multiple comparisons was applied. To analyze possible correlations between DIR direction, focus region, cup size or tidal volume and ROI volume difference or DSI, Generalized Estimating Equations (GEEs) were calculated. They are a semi-parametric regression method, that is useful to analyze correlated or clustered data, especially when measurements are not independent, e.g., multiple measurements from the same subject [26]. Separate GEE models were fitted for DSI and percentage volume difference as dependent variables. DIR direction, focus region, cup size and tidal volume were entered as independent variables. A normal distribution with identity link function and exchangeable working correlation structure was specified, appropriate for continuous repeated measurements from the same subject. P values ≤ 0.05 were considered statistically significant.

## Results

### Descriptive statistical analysis

The mean age of the analyzed patients was 60.92 years (42–87, IQR = 12.5y). Analysis of the breast cup size showed that fourteen patients had a breast volume of 399 ml or less, 59 patients had a breast volume greater than 399 ml. The mean breast volume was 661 ml (129.1 ml – 1294.8 ml, IQR = 474 ml). Forty of 73 of the patients (54%) had a surgical clip. The volumes of the contoured mROIs in FB and DIBH are shown in Table 1.

**Table 1:**
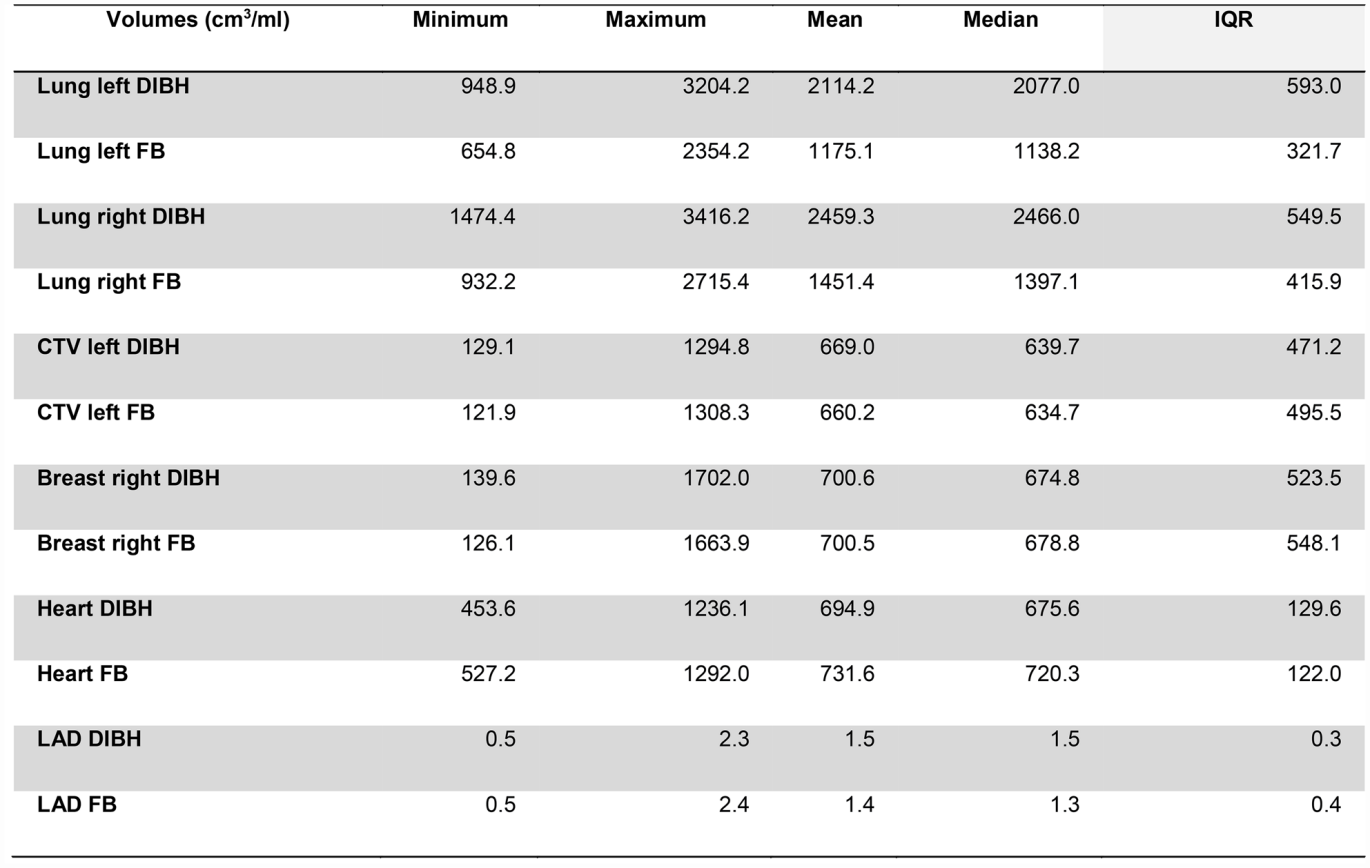
Contoured ROI Volumes in cm3

DIBH = Deep Inspiration Breath Hold, FB = Free Breathing, CTV = Clinical Target Volume, LAD = Left Anterior Descending Artery; IQR = Interquartile Range

Mean total lung volume was 2661.3 ml (1644.9 ml – 5069.6 ml, IQR = 822.3 ml) in FB and 4586.1 ml (2423.3 ml – 6337.7 ml, IQR = 1239.8 ml) in DIBH. The mean calculated tidal volume was 1939.9 ml (249.8 ml − 3070.3 ml, IQR = 976.2 ml). Focusing on specific regions improved registration, while using the entire dataset resulted in average quality. Based on DSI analysis, focusing on specific regions improved geometric overlap within the focus region itself, but was associated with reduced DSI values for non-focused structures.

### CTV - analysis of volume differences observed with DIR

#### ROI transformation from the FB-CT to the DIBH-CT dataset

**Fig. 2:**
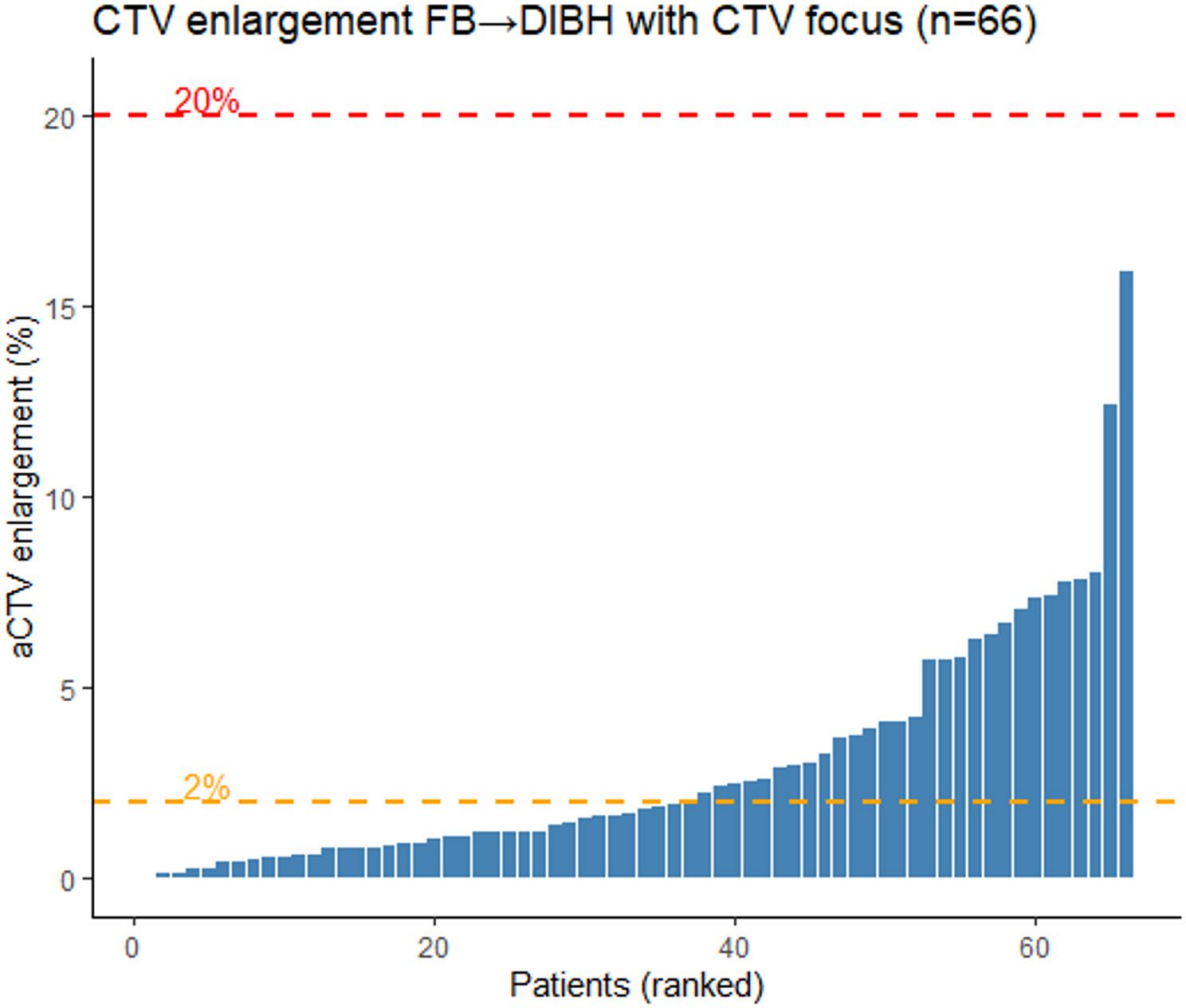
Waterfall plot illustrating the magnitude of aCTV enlargement in FB→DIBH registration with CTV focus. Cases are ranked by enlargement magnitude. Dashed lines indicate 2% and 20% thresholds for clinical reference

Without a focusing region set during image registration, and with the direction and ROI transformation from FB-CT to DIBH-CT dataset, 69% (51 out of 73 cases) showed larger aCTVs compared to manually contoured mCTVs. When the CTV was used as the focusing region, 90% (66 of 73 cases) resulted in larger aCTVs. The mean enlargement in these 66 cases was 2.95% (SD 3.07%, range 0.01–15.90%). 36 cases (55%) showed modest enlargement below 2%, while 14 cases (21%) showed enlargement above 5%. No case exceeded 20% enlargement (Fig. 2). When surgical clips were used as the focusing region in available cases (*n* = 40), 65% (26 of 40 cases) showed larger aCTVs than manually contoured mCTVs.

#### ROI transformation from the DIBH-CT to the FB-CT dataset

Changing the registration direction and using no focusing region resulted in 54 smaller and 19 (26%) larger aCTVs compared to manually contoured mCTVs. Focusing on the CTV itself resulted in 56 cases (76%) with larger aCTVs. However, using surgical clips as the focus resulted in only seven larger (17%) aCTVs. Table 2 shows differences in percentages for the CTV.

**Table 2:**
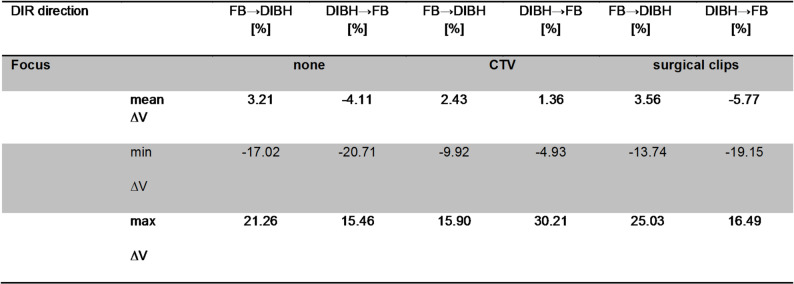
ROI volume for the CTV, differences in percentage

DIBH = Deep Inspiration Breath Hold, FB = Free Breathing, CTV = Clinical Target Volume, DIR = Deformable Image Registration. The → arrow indicates the DIR direction

### OAR - analysis of volume differences observed with DIR

Using DIR with registration direction FB-CT – DIBH-CT resulted 51 larger (69%) heart ROIs (aHeart) when no focus region was set and in 32 larger (43%) aHearts when the heart ROI was used as the focus region. Changing the direction of the image registration process from DIBH CTs to the free breathing CTs resulted in 17 larger (23%) aHeart ROIs when no focus region was set, and in 67 larger (91%) aHeart ROIs when the heart was set as the focus.

### Dice similarity index – DSI

**Fig. 3:**
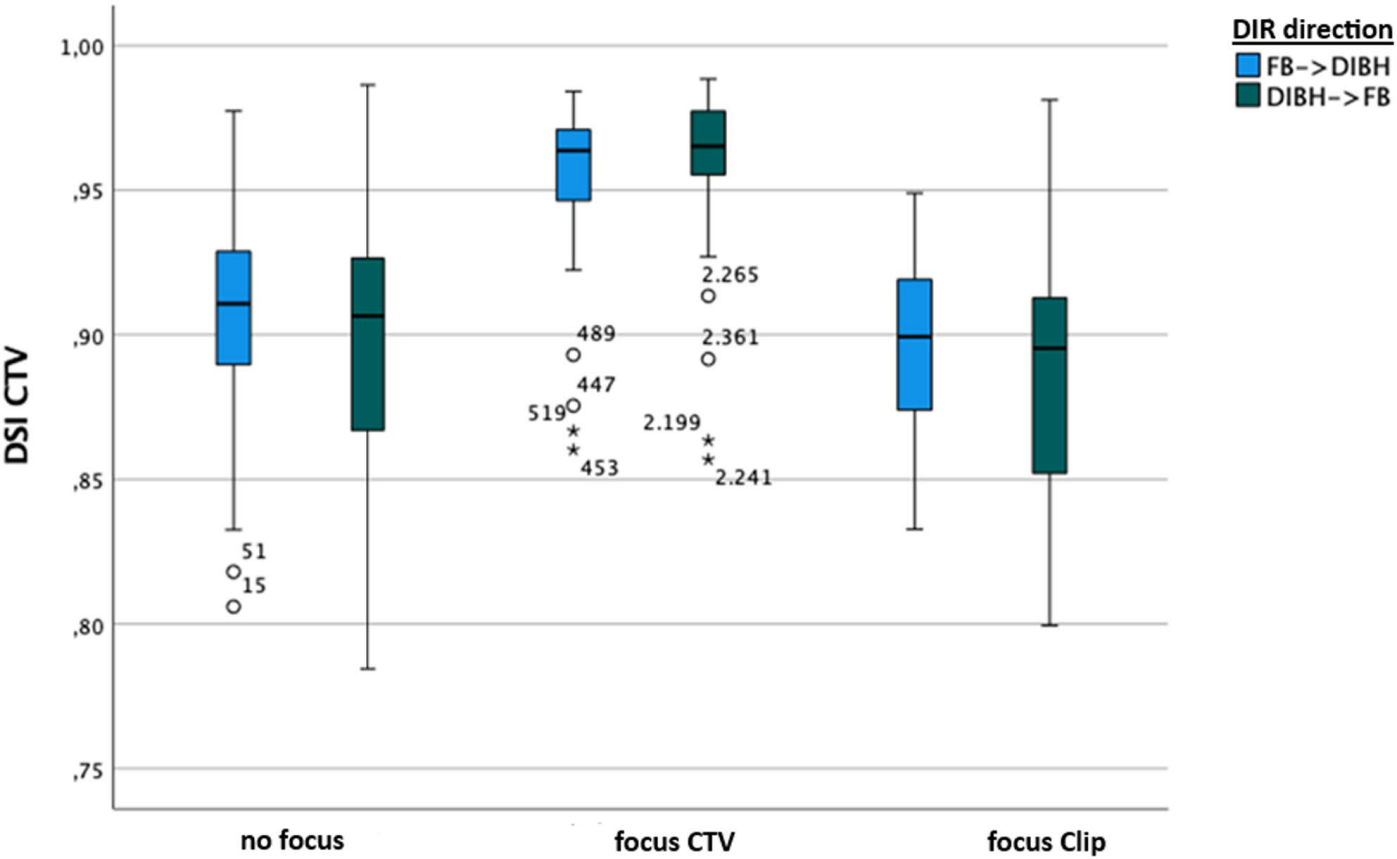
DSI box plot for ROI CTV depending on focus and DIR direction. Outliers are labeled with patient identification numbers for individual case review and quality assessment. FB = free breathing, DIBH = Deep Inspiration Breath Hold. The → arrow indicates the DIR direction

**Fig. 4:**
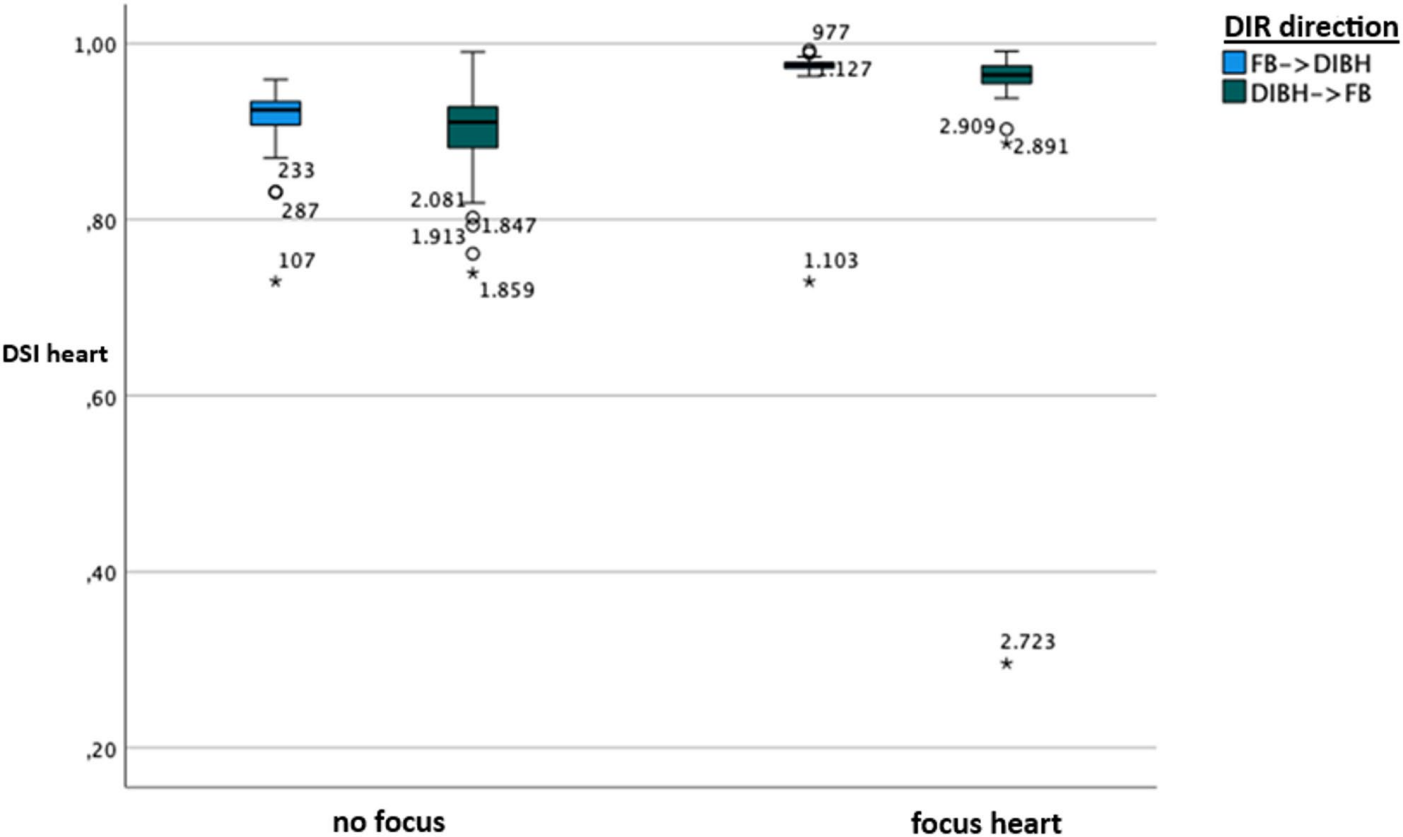
DSI box plot for ROI heart depending on focus and DIR direction. Outliers are labeled with patient identification numbers for individual case review and quality assessment. FB = free breathing, DIBH = Deep Inspiration Breath Hold. The → arrow indicates the DIR direction

The DSI values as a function of the focus region used are shown in Figs. 3 and 4:

For the CTV, DIR from FB to DIBH achieved an average DSI of 0.91 (range: 0.81–0.98) without a focus region, improving to 0.96 (range: 0.86–0.98) with the CTV as the focus region. Using a surgical clip as a focus region resulted in a DSI of 0.9 (range: 0.83–0.95). DIR from DIBH to FB had an average DSI of 0.89 (range: 0.78–0.99) without a focus region, improving to 0.96 (range: 0.86–0.99) with the CTV as the focus region, and 0.89 (range: 0.80–0.98) with a surgical clip as the focus region.

The DSI values as a function of the focus region used are shown in Table 3. It should be noted that the DSI values in this represent averages across both registration directions, which may obscure individual-level variability that is evident in Figs. 3 and 4.

**Table 3:**
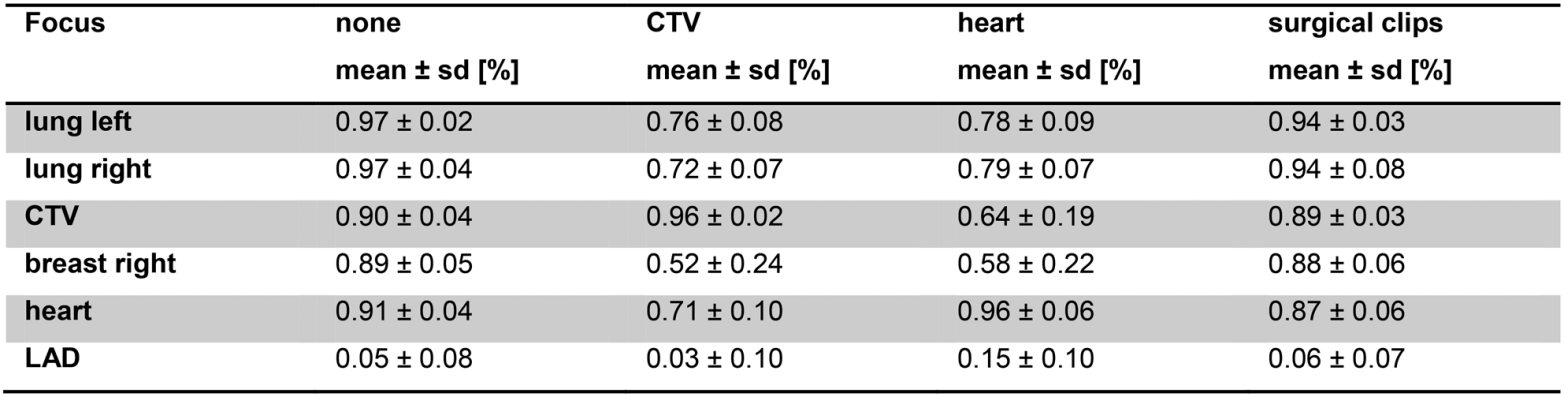
Dice Similarity Index (DSI) values (mean ± SD [%]) by focus region, averaged across both DIR directions (FB→DIBH and DIBH→FB)

sd - standard deviation, CTV - Clinical Target Volume, LAD - Left Anterior Descending Artery

For the heart ROI, DIR from FB to DIBH yielded an average DSI of 0.92 (range: 0.73–0.99) without a focus region, improving to 0.97 (range: 0.73–0.99) with the heart as the focus region. DIR from DIBH to FB gave an average DSI of 0.9 (range: 0.74–0.99) without a focus region, improving to 0.95 (range: 0.3–0.99) with the heart as the focus region.

Overall, focusing on the respective ROI improves its DSI, but adversely affects non-focused ROIs. For example, focusing on the heart improves the DSI of the heart itself and the LAD, but adversely affects the DSI of the lungs, right breast and CTV.

### Inferential statistical analysis

To analyze possible correlations between DIR direction, focus region, cup size or tidal volume and ROI volume difference or DSI, GEEs were calculated, with p values ≤ 0.05 considered statistically significant. DIR direction had no significant effect on CTV volume difference (*p* = 0.84). Setting the CTV as the focus region increased the volume difference by 7.34% (*p* < 0.001). GEE analysis of predictors of CTV volume difference magnitude of enlargement in CTV-focused FB→DIBH registrations showed that larger cup size was associated with smaller differences (β=−3.15%, *p* < 0.001) and tidal volume with larger differences (β = 0.0006% per ml, *p* = 0.006). Registration direction did not significantly affect the magnitude (*p* = 0.121). Using CTV as focus also increased volume difference compared to a clip (8.08%, *p* < 0.001), but no significant result was found between no focus region and a clip (*p* > 0.05). Cup size had no significant effect on CTV volume difference across all focus regions (*p* > 0.05). Similarly, DIR direction had no significant effect on heart volume differences between mHeart and aHeart (*p* > 0.05). Using the heart as the focus region resulted in a 3.14% smaller volume difference compared to no focus region (*p* < 0.05). Cup size had no significant effect on heart volume differences (*p* > 0.05). FB to DIBH fusion significantly increased CTV DSI (0.018, *p* < 0.001), indicating better overlap. CTV as a focus region improved DSI compared to no focus (*p* < 0.001) and compared to using a clip (*p* < 0.001). Tidal volume had an effect on DSI (*p* < 0.005), improving it by 0.05 per 100 ml tidal volume.

DIR with direction FB to DIBH resulted in a 0.018 higher DSI for the heart (*p* < 0.001). Using the heart as the region of focus improved the DSI by 0.055 (*p* < 0.001). Smaller cup size sizes were associated with a higher heart DSI (0.014 points higher, *p* < 0.005). Increasing tidal volume had a negative effect on heart DSI, decreasing it by 0.002 per 100 ml inhaled (*p* < 0.007).

## Discussion

Our retrospective planning analysis of DIR is one of the largest, to our knowledge. The work of Xie et al. on DIR in left-sided breast cancer was based on six and 26 patients [27].

The majority of the published literature focused on DIR is primarily concerned with the evaluation of radiotherapy treatment plans, with only a small proportion investigating volume changes. Table 4 provides a comprehensive overview of selected studies on this topic demonstrating the representativeness of our cohort rather than directly comparing absolute volumes across studies, which are expected to vary due to differences in patient populations and contouring guidelines.

**Table 4:**
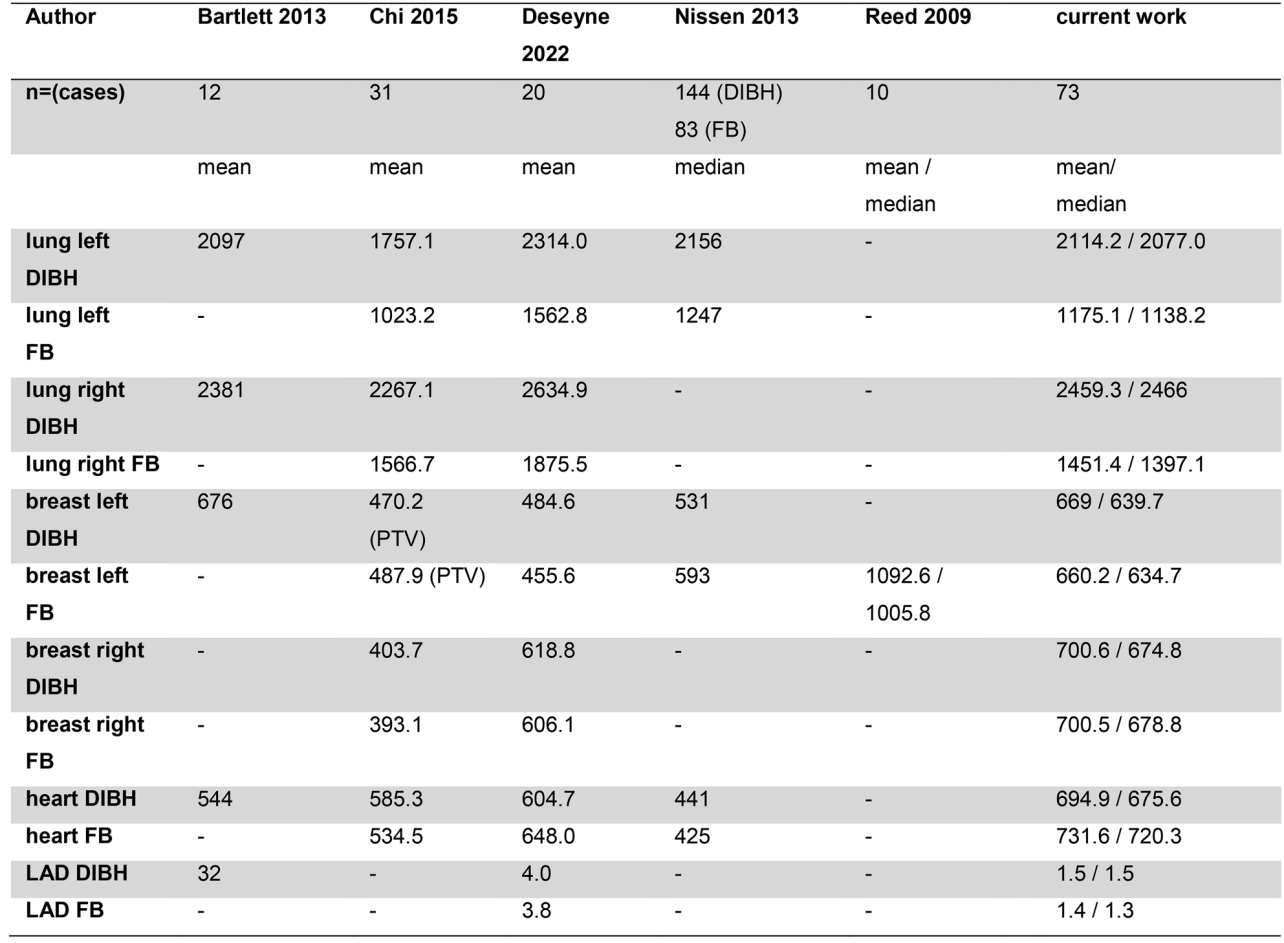
Volumes of ROIs in published scientific work dealing with DIR in left sided breast cancer; volumes are given in cm3/milliliters

DIBH = Deep Inspiration Breath Hold, FB = Free Breathing, LAD = Left Anterior Descending Artery, PTV = Planning Target Volume

A notable strength of our study lies in its more comprehensive analysis of volumes, as most papers predominantly examine one ROI (the heart, left lung and left breast). It’s also worth noting that the published literature on deep inspiration breath-hold studies use different contouring guidelines. In our study, we used the ESTRO guideline for contouring. Bartlett et al. contoured the clinical target volume using visible breast tissue [28]. Chi et al. used visible breast tissue and the ICRU 83 guidelines [29], while Nielsen et al. used the DBCG-guidelines, which are similar to the ESTRO guideline [30]. The use of only visible breast tissue for contouring the CTV should be treated with caution, as different studies have shown large differences in the superior and lateral parts due to the lack of clear anatomical boundaries [31]. In our work, the volume of the CTV in DIBH and FB did not differ very much, which was expected. The chest excursion during the respiratory cycle moves the heart in an inferior and posteriordirection, which results in sparing heart dose, but the mammary gland tissue itself shows little change [32]. The measured CTV in our cohort was 669 ml, which is consistent with the findings of McGhee et al. In their study, 643 ml was measured, although they used a custom-designed water displacement device instead of CT-graphic measurement [19]. As mentioned above, different studies used different guidelines for CT-graphic contouring. The heart volumes shown in the work of Nissen et al. appear to be low, which is due to the fact that the ROI heart was contoured without including the pericardium and coronary arteries [33]. This is insufficient with regard to evaluation of long-term toxicities, such as pericarditis or coronary artery disease [6, 34, 35]. DIBH showed smaller heart volumes than FB (compare Table 1). One possible explanation is a volume shift during inspiration towards the lungs and their blood pool [32]. Volume differences and DSI varied with focusing region use. The CTV and heart achieved highest DSI values with specific focusing. The relatively large volumes and well-defined boundaries of both structures likely contribute to their higher DSI values, consistent with published observations that larger structures with clearer boundaries tend to achieve more favorable DSI scores [23]. Surgical clip as focus showed intermediate outcomes, potentially due to its CTV association. This finding has particular relevance for clinical practice. In sequential boost techniques, clip-focused DIR may suffice for tumor bed propagation alone. However, in simultaneous integrated boost (SIB) protocols, where the whole breast CTV and boost volume are irradiated concurrently, clip-focused registration produces suboptimal whole-breast CTV alignment, consistent with our observed DSI of 0.89 for CTV with clip focus compared to 0.96 with CTV focus. This trade-off is well recognized in daily clinical practice and underscores that no single focus region simultaneously optimizes all clinically relevant structures in SIB planning.

The systematic enlargement of aCTVs when using CTV focus (observed in 90% of FB→DIBH cases) was further characterized by applying GEEs. The mean enlargement was modest at 2.95% (SD 3.07%), and the majority of cases showed enlargement below 2%. However, enlargements exceeding 5% were observed in 21% of cases, highlighting the necessity of mandatory manual review. The magnitude of enlargement was significantly associated with cup size (*p* < 0.001) but not with registration direction (*p* = 0.121), suggesting that patient anatomy rather than acquisition protocol drives this systematic behavior. This represents a novel finding that, to our knowledge, has not been previously reported in the literature.

A focusing region acts as a control structure, defining deformation vectors within its boundaries. This highlights the role of focusing regions in guiding the deformation process for improved accuracy [20]. The improvement of the DSI of DIR using Raystations^®^ algorithm was also shown by Romanò et al. [36]. In this work, GEEs were calculated for the ROIs CTV and the heart. The impact of using a focusing region on the entire image is beyond the scope of this analysis. Further research is needed to investigate the impact on the complete dataset before implementing it in daily practice, as dose recalculation is based on the whole dataset. No link between cup size and CTV/breast DSI was found, differing from Anders et al. who used atlas-based autosegmentation. They found a higher DSI for the CTV in patients with a larger cup size. Contour shape seems to influence DSI more than cup size [37]. Inspiration depth significantly affected DSI. CTV’s DSI increased, while heart’s DSI decreased with deeper inspiration. One possible explanation may be that the increased air volume during inspiration leads to better visualization of the heart. It should be noted that in this study the Focus ROI function was used, which optimizes registration accuracy within the selected region through localized Gaussian smoothing without imposing an active penalty term on the deformation field. RayStation’s alternative Controlling ROI function adds an explicit penalty term to force alignment of the selected structure, which would speculatively result in even larger deviations in non-focused structures. The trade-offs reported in this study may therefore represent a conservative estimate, though this hypothesis warrants future investigation. A strength of our study is that DIR was investigated in two datasets of the same patient among 73 consecutive patients. Published literature that covers DIR often uses atlas based autosegmentation between a template and a new image dataset. Choosing a template that fits the BMI and cup size did improve the results [37, 38]. Our method circumvents the challenges related to varying body mass indices and cup size, as noted by other researchers [16], since the sole shift present at the individual patient level is chest excursion [19]. A drawback of our approach, when applied in real-world scenarios, is the necessity for thorough evaluation and manual adaptation of a structure set each time, stemming from the lack of a perfect atlas. Nevertheless, advancements in DIR algorithms may eventually enable the utilization of template patients, as exemplified by Fontanilla et al. [39]. With DSI values consistently above 0.75 for the main target structures, our results meet contemporary quality standards for DIR validation [40]. Our work is in concordance with the published literature dealing with DIR [37]. It’s noteworthy to highlight that this observation does not extend to the ROI LAD, as it consistently yielded low DSI values irrespective of the presence of a focusing region. This result aligns with existing literature in the field, which demonstrates that DIR is more accurate with structures possessing clearly defined boundaries. Consequently, smaller structures, which often lack such distinct boundaries, tend to exhibit lower DSI values [23]. An improved overlap can be reached by adding a one-centimeter margin [38]. DIR itself can reach sufficient image overlaps, as shown by Simoes et al., which did compare autosegmented structures with manually adjusted structures, with a resulting DSI of 0.95 [41]. When LAD dose evaluation is clinically relevant, heart-focused registration is recommended as it achieved the highest LAD DSI (0.15 vs. 0.05 without focus). Given that DIR-propagated LAD contours consistently yielded DSI values of 0.15 — significantly below the inter-observer variability of manual LAD delineation (DSI 0.34+/-0.17) [42] — full re-delineation rather than mere review is required in clinical practice, regardless of focus region selection.

To the best of our knowledge, besides our own data, the study by Xie et al. is the only published work that addresses structure-based DIR [27]. Their research focused on the value of masking breast tissue and employing predefined focus landmarks to address the discontinuity between pre- and postoperative image datasets. Their findings highlighted the absence of anatomical landmarks as a limitation. In our study, we employed surgical clips as landmarks. Our results, indicating that integrating structure-based information with Hounsfield units-based image registration enhances the registration process, align with published research [27, 36]. For Raystation TPS^®^, ANACONDA performances can be influenced with controlling ROIs. Romano et al. described a saturation level of DIR performances when four controlling ROIs are included [36]. As described, we used the DSI and volume differences to calculate the precision between the manually contoured mROI and by DIR automatically generated aROI. Inverse deformation, which translates DIR back after the first registration process in the original dataset, would be another method to investigate the quality of a TPS DIR [15, 43].

The generalizability of our findings is limited to the RayStation^®^ ANACONDA algorithm. While the general principle that focus region selection creates trade-offs between local and global accuracy may apply to other hybrid DIR algorithms, the specific quantitative relationships observed in this study should not be extrapolated to other implementations without further validation. The methodological framework presented here consisting of bidirectional evaluation, systematic focus region variation, and GEE-based analysis of patient-specific predictors can however serve as a template for analogous evaluations of other DIR implementations.

A limitation of our study for clinical practice is the sole geometrical evaluation of the ROIs CTV and heart. Volume differences and volume overlap measured by DSI cannot provide conclusive implications about clinical relevance. Spatial dose-volume information needs to be collected and evaluated in a future step to establish clinical significance, but this was beyond the scope of our current work. A further methodological limitation is the exclusive use of the Dice Similarity Coefficient for DIR validation. Complementary metrics such as Hausdorff distance or surface distance measures would provide a more comprehensive assessment of boundary discrepancies and local registration errors, and should be incorporated in future studies. Nevertheless geometric inaccuracies in DIR-generated contours can in principle have dosimetric implications, as demonstrated by Simoes et al., who showed that incorrect volumes may translate into relevant underdosage or higher lung doses — underscoring the need for dosimetric validation in future work [41]. DIR can be a tool in the armamentarium of the radiation oncologist for specific questions, and reaches congruent results for the focused sub-volume, whereas with regard to the whole volume, manual adaptation has to be done. However, our findings reveal that DIR implementation faces significant practical challenges. The necessity for structure-specific parameter optimization, combined with the trade-offs between focused and non-focused ROIs, limits the efficiency gains that DIR was intended to provide. Manual review and adjustment remain essential regardless of parameter selection.

Based on our findings and clinical experience, we offer the following implementation guidance as expert opinion, acknowledging that patient-level decision criteria require future dosimetric validation: (1) If cardiac dose evaluation is the primary concern, heart-focused registration should be used, with mandatory manual review of CTV contours. (2) If target volume assessment is the primary concern, CTV-focused registration is preferred, with mandatory manual review of cardiac structures. (3) Registration direction (FB→DIBH vs. DIBH→FB) may be standardized within institutions as no clinically significant difference was found. (4) Given that manual review remains essential regardless of parameter selection, DIR should be positioned as a tool for specific clinical questions rather than a general workflow accelerator.

Several limitations of this study should be acknowledged. First, the evaluation is purely geometrical; dosimetric validation remains essential future work. Second, findings are specific to RayStation’s ANACONDA algorithm and may not generalize to other DIR implementations. While open-source DIR algorithms exist [44], their use in clinical practice is precluded by medical device regulations, which require validated and certified software for clinical decision-making. This makes vendor-specific evaluations, such as the present study, the primary means of assessing DIR performance in routine clinical workflows. Third, focus regions were evaluated individually rather than simultaneously, which may differ from typical clinical workflows. Fourth, the cohort comprised exclusively patients without nodal irradiation, limiting generalizability to more complex target configurations. It should be noted that our cohort exclusively comprised patients without indication for nodal irradiation, so the CTV represented a single anatomical structure (i.e. the whole left breast). Future studies should investigate whether the observed trade-offs are exacerbated when simultaneously propagating multiple anatomically distinct sub-volumes, such as CTVs required in patients with nodal irradiation. Dosimetric validation on a representative subset remains an essential next step, as geometric accuracy alone cannot fully characterize clinical relevance of DIR-generated contours. Another possible limitation is, that the quality of rigid registration was evaluated visually rather than through quantitative measures. However, this approach reflects standard clinical practice, where rigid registration is routinely assessed by visual inspection of anatomical landmarks. Furthermore, separate analysis of cases with and without surgical clips would provide additional insight into the role of anatomical landmarks in DIR accuracy. Nevertheless, it should be noted that findings related to clip-focused registration are applicable only to the 40 of 73 patients (55%) who had surgical clips present. Generalizability to patients without clips remains untested. Additionally, deformation vector fields were not available for analysis, precluding a definitive algorithmic explanation for the systematic CTV enlargement observed with CTV-focused registration.

## Conclusion

This study demonstrates that DIR performance in breast radiotherapy planning is critically dependent on user-defined parameter selection. Focus region choice creates an inherent trade-off between local accuracy and global registration quality, with no single configuration simultaneously optimizing all structures. Registration direction showed no clinically significant effect on volume metrics, allowing institutional standardization. Patient-specific factors such as tidal volume and breast volume influence DIR accuracy in structure-dependent ways. Based on these findings, structure-specific focus region selection with mandatory manual review of non-focused structures is recommended for clinical implementation. DIR should be positioned as a targeted tool for specific clinical questions rather than a general workflow accelerator, with dosimetric validation representing an essential direction for future work. While obtained using RayStation’s ANACONDA algorithm, these findings highlight the importance of systematic parameter evaluation for any hybrid DIR system. 

## Data Availability

The dataset is available from the corresponding author upon reasonable request.
